# Sex differences in dementia: on the potentially mediating effects of educational attainment and experiences of psychological distress

**DOI:** 10.1186/s12888-020-02820-9

**Published:** 2020-09-04

**Authors:** Caroline Hasselgren, Hans Ekbrand, Björn Halleröd, Madeleine Mellqvist Fässberg, Anna Zettergren, Lena Johansson, Ingmar Skoog, Lotta Dellve

**Affiliations:** 1 Department of Sociology and Work Science, University of Gothenburg, Box 720, 405 30 Gothenburg, Sweden; 2 Department of Psychiatry and Neurochemistry, Institute of Neuroscience and Physiology, Sahlgrenska Academy, Centre for Ageing and Health - AgeCap, University of Gothenburg, Mölndal, Sweden

**Keywords:** Dementia, Gender inequity, Education, Psychological distress, Structural equation modelling, Prospective population study

## Abstract

**Background:**

Old-age dementias are known to disproportionally affect women as well as individuals with low educational attainment. The higher lifetime risk of dementia among women is usually attributed to their longer life expectancy. However, the impact of sex, and subsequent gender inequity, is likely to be more multifaceted than this explanation implies. Not least because of historical inequities in access to education between the sexes and the gender and socio-economic gradients in risk factors such as stress, depression and social isolation. Consequently, the present study sought to test whether differences in educational attainment and experiences of general psychological distress mediate the association between female sex and dementia.

**Methods:**

The study utilizes data obtained through the *Gothenburg H70 Birth Cohort Study* and the *Prospective Populations Study on Women* (*n* = 892). Data were analysed using Confirmatory Factor Analysis (CFA) and Structural Equation Modelling (SEM) with Weighted Least Squares Means and Variance adjusted (WLSMV) estimation. General psychological distress was indicated by a latent variable and constructed from five manifest items (previous depression, stress, self-esteem, chronic loneliness and satisfaction with social situation) that were all measured at baseline*.*

**Results:**

While the results could not corroborate that education directly mediates the effect of sex on dementia, level of distress was predicted by both female sex (0.607, *p* < .001) and education (− 0.166, *p* < .01) and, in turn, shown to be significantly associated with dementia (0.167, *p* < .05), also after controlling for confounders. When time from baseline to diagnosis was increased through sequential exclusion of dementia cases, the effect of distress on dementia was no longer significant.

**Conclusion:**

The overall findings suggest that social (dis) advantage predicts general psychological distress, which thereby constitutes a potential, and rarely acknowledged, pathway between female sex, education, and dementia. They further underline the importance of attending to both education and distress as ‘gendered’ phenomena when considering the nature of their associations with dementia. However, the possibility of reverse causality bias must be acknowledged and the need for longitudinal studies with longer follow-up stressed.

## Background

Due to worldwide demographic changes in population age composition, the number of people living with dementia is increasing and currently regarded as one of the major public health issues of our times [1, 2]. The most common cause of dementia is Alzheimer’s disease (AD). It is estimated to account for 50–70% of all cases, and the majority of people living with dementia have multimorbidity involving Alzheimer-related changes in combination with other pathologies, particularly cerebrovascular changes [1, 3–7]. The major genetic risk factor for AD is the apolipoprotein E (*APOE*) ɛ4 allele, which is a non-causative mutation known to increase disease risk by between three and 15 times [8, 9]. Old-age dementias, particularly AD, are further known to disproportionally affect women and individuals with lower educational attainment [1, 10, 11]. With respect to sex, estimates of differences in prevalence and incidence rates of AD and other dementias are somewhat conflicting and seem to vary by type, age group and geographical region [1, 12–17]. Nevertheless, compelling evidence suggests that women have a greater lifetime risk of developing AD or any dementia [16, 18, 19]. For instance, at age 65, 1 in 7 men are projected to develop the disease during their remaining life span, which yields a lifetime risk of approximately 14%. The corresponding estimate for women is 20% [18].

The higher lifetime risk of dementia among women has previously been attributed mainly to their longer life expectancy, but the impact of sex and subsequent gender inequity on dementia risk is likely to be more multifaceted than this explanation implies [16, 20, 21]. First and foremost, it is well known that systematic inequities in access to education – one of the most established risk factors for both AD and vascular dementia (VaD) [10, 22] – have existed between men and women in previous generations. Educational differences in dementia are often explained by the cognitive reserve hypothesis, which emphasizes the role of intellectually stimulating activities is preventing symptoms of degenerative brain changes [23, 24]. However, education is also a strong predictor of individuals’ future labour market positions, and hence of their socio-economic status (SES) [25, 26]. Today, it is well established that many potential (e.g., depression) and influential (e.g., social isolation and stress) dementia risk factors follow both gender and/or socio-economic gradients [10, 27]. Thus, it appears plausible that the relationship between female sex and dementia could be mediated by, inter alia, differences in educational attainment as well as ensuing symptoms of distress, especially considering that structural (dis) advantage accumulates over time as well across life domains [28–30].

Even though consensus concerning the direction of causality does not yet seem to prevail [31, 32], depression has attracted attention as a possible dementia risk factor because of its influence on, e.g., stress hormones, neuronal growth, and hippocampal volume [10, 33]. While some studies have indicated that it should primarily be considered a prodrome [34, 35], others suggest that mid-life depression, or recurrent depressive episodes, are actually independent risk factors [36, 37]. Moreover, a number of systematic reviews conclude that it might actually be both ways, i.e., while earlier-life depression constitutes a potential risk factor, late-life depression is likely to be a preclinical sign [38–40]. In any case, depressive disorders are known to be more widespread among women as well as among individuals with lower SES [41–44]. Likewise, social support appears to follow a social gradient, in favour of individuals with higher SES [45, 46], and it is generally agreed that there is an inverse relationship between social isolation and mental health [47, 48]. While social isolation, just like depression, could be both a symptom or prodrome of dementia, emerging evidence suggest that it is in fact an independent risk factor [10, 49, 50]. Finally, long-standing exposure to unfavourable, material and/or psychosocial conditions deriving from, e.g., gender or socio-economic inequity is assumed to elicit sustained stress reactions. Thus, stress is usually considered to be one of the main pathophysiological pathways linking social (dis) advantages to health/longevity [27, 51]. In line with this, women are known to suffer a greater burden of stress and associated symptoms in comparison to men [52–54], and numerous studies have found various types of stress to be associated with dementia [55–60]. These associations are often considered attributable to the fact that stress increases cardiovascular risk, such as in hypertension [61, 62], and heightens levels of glucocorticoid hormones in the blood, which can, e.g., cause damage to the hippocampus [63–65].

In light of recent insights concerning the multitude of ways in which sex/gender can affect disease risk, more research targeting the specific mechanisms involved in disease development is of the utmost importance [16, 20, 66]. Consequently, the present study seeks to shed further light on the complex relationship between dementia and social inequity by exploring the extent to which the association of sex with dementia is mediated by differences in levels of educational attainment and experiences of general psychological distress. The more specific hypotheses read as follows:

- H_1_: The effect of female sex on dementia is mediated directly by differences in level of educational attainment, also after controlling for age and *APOE* ε4 allelic status.
- H_2_: The effect of female sex on dementia is mediated directly, as well as indirectly via education, by level of general psychological distress, also after controlling for age and *APOE* ε4 allelic status.

## Methods

### Study sample

The present study utilizes data obtained through two longitudinal studies from Gothenburg, Sweden: the H70 Birth Cohort Study and the Prospective Populations Study of Women (PPSW). All participants were sampled from the Swedish population register and systematically selected on the basis of birthdate. Of 1572 possible study participants living in Sweden on September 1, 2000, 1495 were eligible (i.e., Swedish speaking, not deceased and still living in Sweden at the time of the examination), and 1019 consented to participate in the psychiatric examination (response rate 68.2%). This sample consists of 229 men (22.5%) and 790 women (77.5%). The women were born in 1908, 1914, 1918, 1922 and 1930, and all men were born in 1930. Among these, 923 (90.6%) participants agreed to donate blood for genetic analyses. Follow-up examinations were carried out in 2005–06 (*N* = 724) and 2009–10 (*N* = 529). A more detailed description of the baseline sample can be found elsewhere [67]. Because analyses of mediation presuppose temporal precedence [68], individuals who were diagnosed with dementia prior to, or at, baseline were excluded (*n* = 94). We also excluded individuals who developed dementia during the first year after the baseline examination (*n* = 2) or had a baseline Mini Mental State Examination (MMSE) score of less 24 (out of max. 30) points (*n* = 31) [69–71]. Foremost, this was done in order to circumvent possible bias in relation to the factor indicators since cognitive impairment is likely to affect the understanding of, and/or responses given to, survey questions. Another reason for excluding these individuals was that they constitute yet another potential source of reverse causality bias. The final sample consisted of 892 individuals and informed consent was acquired from all participants. The studies were approved by the regional Ethics Review Board for medical research in Gothenburg [67].

### Neuropsychiatric examinations, diagnoses and genotyping

The clinical examinations were conducted at an outpatient department or in the participants’ homes and comprised comprehensive social, functional, physical, neuropsychiatric and neuropsychological assessments. The semi-structured neuropsychiatric examinations were performed by trained psychiatric research nurses and included ratings of common symptoms and signs of dementia (e.g., assessments of memory, orientation, general knowledge, apraxia, visuospatial function, understanding proverbs, following commands, naming ability and language), and have been described in more detail elsewhere [72, 73]. In addition, a semi-structured telephone interview with a close informant was performed and comprised, e.g., questions regarding changes in behaviour and intellectual function, psychiatric symptoms, activities of daily living, and, in cases of dementia, age of onset and disease course [67, 74]. Dementia was diagnosed by geriatric psychiatrists using the Diagnostic and Statistical Manual of Mental Disorders, 3rd Edition Revised (DSM-III-R) [75]. The diagnoses were based on symptoms rated during the neuropsychiatric examinations as well as on information from the close informant interviews [72, 73]. For individuals lost to follow-up, incident dementia cases (until 2012) were diagnosed on the basis of information from medical records, evaluated by geriatric psychiatrists, or from the Swedish Hospital Discharge Register [72]. The main outcome variable ‘Dementia t_1_’ indicates whether or not an individual developed any type of dementia during the period 2002–12 (Table 1). Blood samples were collected and the SNPs (single nucleotide polymorphisms) rs7412 and rs429358 in *APOE* (gene map locus 19q13.2) were genotyped using the KASPar® PCR SNP genotyping system (LGC Genomics, Hoddesdon, Herts, UK) or by mini-sequencing, as previously described in detail [76]. Genotype data for these two SNPs were used to define ε2, ε3, and ε4 alleles. Because ε4 is the only allele associated with an increased risk of AD, the subsequent analyses controlled only for carriership of this variant (Table 1). Consequently, since current evidence concerning the combined effect of the ε2/ε4 genotype on AD risk is scarce [77], and the relative frequency of ε2/ ε4 carriers in the present sample was small (approx. 1.5%), they were not excluded from the analyses.

**Table 1:** Baseline characteristics of the study population

	All	Females	Males	Sig. for difference
	n (%) | *M* (SD)	n (%) | *M* (SD)	n (%) | *M* (SD)	*p*
Sex	892 (100)	672 (75.3)	220 (24.7)	–
Age at baseline	74.5 (5.4)	75.9 (5.6)	70.5 (0.2)	***
Presence of APOE ɛ4^a^	239 (27.8)	176 (27.4)	63 (28.9)	n.s.
Educational attainment^b^				***
Primary	529 (60.6)	408 (62.4)	121 (55.3)	–
Lower secondary	207 (23.7)	168 (25.7)	39 (17.8)	–
Secondary/university	137 (15.7)	78 (11.9)	59 (26.9)	–
Previous depression^c,^ *	260 (29.3)	216 (32.3)	44 (20.0)	***
One or more period(s) of longstanding stress^d^	369 (47.3)	273 (48.3)	96 (44.0)	n.s.
Satisfaction w. social situation (1–7)^e^	2.6 (1.2)	2.7 (1.2)	2.5 (1.1)	*
Chronic loneliness^f^	106 (12.1)	91 (13.7)	15 (6.8)	***
Self-esteem (1–7)^g^	3.1 (1.1)	3.3 (1.2)	2.7 (1.0)	***
Developed dementia 2002–12 (t_1_)	144 (16.1)	124 (18.5)	20 (9.1)	***
Developed dementia 2003–12 (t_2_)	126 (14.4)	111 (16.8)	15 (7.0)	***
Developed dementia 2004–12 (t_3_)	108 (12.6)	95 (14.8)	13 (6.1)	***
Developed dementia 2005–12 (t_4_)	91 (10.9)	78 (10.9)	13 (6.1)	**
Developed dementia 2006–12 (t_5_)	81 (9.8)	70 (11.3)	11 (5.2)	**

Note: Total *N* = 892, * = self-reported. Significance of group differences assed using the Ttwo-sample t-test or Perason’s χ^2^-test, *p < 0.05. **p < 0.01. *** *p* < 0.001. ^a^Information missing for 31 subjects (3.5%). ^b^Information missing for 19 subjects (2.1%). ^c^Information missing for 4 subjects (0.4%). ^d^Information missing for 111 subjects (12.4%). ^e^Information missing for 52 subjects (5.8%). ^f^Information missing for 6 subjects (0.7%). ^g^Information missing for 53 subjects (5.9%)

### Operationalizations

#### Assessment of general psychological distress

In the present analyses, *General psychological distress* was indicated by a latent variable (for further details, see below)*.* This variable was constructed from five manifest items that were all measured at baseline (Table 1). The first, *Previous depression (yes/no)*, is self-reported and indicates whether or not an individual had suffered from depression prior to the baseline examination. The second item, *Have experienced one or more period(s) of stress in life (yes/no),* was constructed from the following survey question: ‘Have you experienced any period of stress (one month or longer) in relation to circumstances in everyday life, such as work, health or family situation? By stress we mean feelings of irritability, tension, nervousness, fear, anxiety or sleep disturbances’. The following response alternatives were available: *‘*0: I have never experienced any period of stress’; 1: ‘I have experienced period(s) of stress more than 5 years ago’; 2: ‘I have experienced one period of stress during the past 5 years’; 3: ‘I have experienced several periods of stress during the past 5 years’; 4: ‘I have experienced constant stress during the past year’; or 5: ‘I have experienced constant stress during the past 5 years’. Owing to the nature of these alternatives (they are not mutually exclusive, and respondents were only allowed to specify one answer), individuals were classified into two groups based on their responses: those who had experienced period(s) of longstanding stress (at any time) and those who had not. More specifically, this was done in order to avoid misclassification of individuals who had suffered from stress *both* previously in life as well as during later years. The third and fourth indicator, *Satisfaction with social situation* and *Self-esteem*, were obtained from two items in a battery of questions in one of the baseline questionnaires, where participants were asked to rate their satisfaction in relation to different life domains on an ordinal scale from 1 (‘very good, could not be better’) to 7 (‘very bad’). The fifth indicator, *Chronic loneliness*, was based on the following question: ‘Do you feel lonely?’. The respondents could also specify how long they had been feeling lonely (1. ‘Yes, for more than 5 years’, 2. ‘Yes, for 1-5 years’, 3. ‘Yes, for 0-1 years’). To further limit reverse causality bias, only those who had been feeling lonely for 5 years or longer were coded as ‘yes’.

#### Assessment of education

At baseline, all respondents were asked to specify the level/type of their educational attainment. In cases where information was missing, it was, if available, obtained from the follow-up examinations. Based on these responses, a variable with three values indicating educational level was constructed: *Primary* corresponds to elementary school/vocational school, *Lower secondary* to girls’ school/junior secondary school/folk high school and *Secondary/university* to high school/university [78].

### Statistical analyses

The present paper utilizes confirmatory factor analysis (CFA) and structural equation modelling (SEM) to test the hypothesized relationships [79] between female sex, education, psychological distress (latent construct), and dementia. Compared to commonly used additive indices, latent variables comprise only the covariance between the manifest indicators. The residual variances are estimated separately and can be incorporated into the model, which means that latent constructs are, in a sense, ‘error free’ [80]. The analysis was conducted in two steps. First, CFA was used to estimate a measurement model in order to confirm that the indicator variables in question were linked to the underlying latent construct *General psychological distress*. Second, a full structural model was specified to test the main hypotheses. The full model included both the latent factor and the observed variables, and all coefficients were estimated simultaneously. Finally, because reverse causality is a significant issue in studies dealing with factors that could be risk factors for and/or pre-diagnostic signs of dementia [49, 50, 81, 82], we also conducted a sensitivity analysis. This was done by sequentially excluding dementia cases, starting with those that occurred in closest proximity to baseline, in order to investigate whether identified relationships remain significant at different time points. All descriptive analyses were conducted using STATA 15, and the CFA as well as the structural models were estimated using MPlus version 8.

Because the observed dependent variables are categorical (binary or ordered) and skewed, we used the weighted least squares means and variance adjusted (WLSMV) estimator [83, 84] and report unstandardized probit regression coefficients. Since the WLSMV estimator is computationally limited in handling missingness that has not occurred completely at random (MCAR), missing values were imputed using Bayesian estimation where data is generated from an MCMC (Markov Chain Monte Carlo) simulation and imputed using an unrestricted variance-covariance model. Five data sets were generated and analysed simultaneously in all stages of the modelling procedure, which means that the parameter estimates and standard errors represent averages over the five sets of analyses [85, 86]. Following the imputation of missing values for all dependent variables with missing data, the confirmatory factor analyses were based on the full sample (*n* = 892) (Table 1-2). The structural models were based on data from 861 individuals, as individuals with missing data on independent variables, in our case *APOE* ɛ4 (*n* = 31), were excluded (Table 3). Following standard SEM-procedures, we relied on the following fit indices to evaluate model fit: The Root Mean Square Error of Approximation (RMSEA), the Tucker Lewis Index (TLI) and the Weighted Root Mean Square Residual (WRMR). χ^2^ is also reported, but because it is known to be inflated when *N* is large [79, 83], we followed Iaccobucci [87], who suggests that a χ^2^/df ratio ≤ 3 exhibits reasonable fit. For RMSEA, values below .05 [88] were considered indicative of good fit. With regard to WRMR, a value of < 1 has generally been considered to suggest a well-fitted model [89, 90], even though researchers have been advised not to rely too heavily on this measurement, given that it is sensitive to, e.g., non-normality and larger sample sizes [90]. For TLI, values of above .90 [91, 92] or ‘close to .95’ [93] were judged to indicate of good fit. However, because the stricter values have later been criticized for being too rigid [91], all measures were evaluated concurrently as well as in relation to the theoretical plausibility of the models [79, 83].

**Table 2:** Measurement models

	Hypothesized			Adjusted (final)		
	Factor loading (λ)	95% CI	*p*	Factor loading (λ)	95% CI	*p*
Previous depression	0.611	0.520–0.701	***	0.601	0.475–0.728	***
Chronic loneliness	0.569	0.461–0.677	***	0.696	0.557–0.835	***
One or more period(s) of longstanding stress	0.491	0.384–0.599	***	0.412	0.268–0.555	***
Satisfaction with social situation	0.669	0.599–0.739	***	0.540	0.420–0.660	***
Self-esteem	0.588	0.514–0.661	***	0.428	0.313–0.543	***
Depression_res_ ↔ Stress_res_	–	–	–	0.284	0.161–0.408	***
Self-esteem_res_ ↔ Social situation_res_	–	–	–	0.229	0.127–0.331	***
N	892			892		
χ^2^ (df)	85.969 (5)			6.890 (3)		
TLI	0.767			0.981		
RMSEA	0.135			0.034		
WRMR	1.598			0.388		

Note: Unstandardized WLSMV (probit) estimates, *** *p* < 0.001

**Table 3:** Full structural models

	M1 (conceptual, t_1_)			M2 (final, t_1_)			M3 (final, t_2_)		
	β	95% CI	*p*	β	95% CI	*p*	β	95% CI	*p*
DISTRESS →									
Previous depression	0.605	0.419–0.792	***	0.614	0.426–0.802	***	0.628	0.429–0.827	***
Chronic loneliness	0.782	0.511–1.052	***	0.784	0.513–1.055	***	0.820	0.517–1.124	***
Stress	0.338	0.194–0.481	***	0.345	0.200–0.489	***	0.322	0.183–0.482	***
Satisfaction with social situation	0.724	0.496–0.953	***	0.713	0.491–0.934	***	0.683	0.477–0.773	***
Self-esteem	0.623	0.422–0.825	***	0.616	0.419–0.813	***	0.592	0.412–0.773	***
DISTRESS → Dementia	0.150	− 0.003 – 0.302	†	0.167	0.020–0.314	*	0.090	− 0.057 – 0.238	ns.
Education→ DISTRESS	− 0.164	0.302–0.804	**	− 0.166	− 0.286 – − 0.047	**	− 0.185	− 0.309 – − 0.061	**
Education → Dementia	− 0.022	− 0.160 – 0.115	ns.	–	–	–	–	–	–
Sex → Education	− 0.308	− 0.530 – − 0.087	**	− 0.345	− 0.521 – − 0.170	***	− 0.332	− 0.510 – − 0.155	***
Sex → DISTRESS	0.575	0.302–0.848	***	0.607	0.361–0.852	***	0.631	0.380–0.882	***
Sex → Dementia	0.008	− 0.995 – 1.011	ns.	–	–	–	–	–	–
Sex → Age	5.256	− 12.213 – 18.549	ns.	5.243	1.213–9.273	*	7.452	2.714–12.191	***
Age → DISTRESS	0.006	− 0.013 – 0.025	ns.	–	–	–	–	–	–
Age → Education	− 0.007	− 0.025 – 0.011	ns.	–	–	–	–	–	–
Age → Dementia	0.069	0.044–0.093	***	0.070	0.047–0.093	***	0.063	0.042–0.083	***
*APOE* ε4 → Dementia	0.231	− 0.003 – 0.465	†	0.232	− 0.002 – 0.466	†	0.228	−0.011 – 0.467	†
Depression_res_ ↔ Stress _res_	0.464	0.349–0.579	***	0.461	0.345–0.577	***	0.463	0.344–0.583	***
Self-esteem_res_ ↔ Social situation_res_	0.196	0.046–0.347	***	0.203	0.059–0.348	**	0.223	0.093–0.354	***
N	861			861			843		
χ^2^ (df)	75.521 (26)			73.107 (30)			72.208 (30)		
TLI	0.895			0.921			0.920		
RMSEA	0.047			0.041			0.041		
WRMR	1.016			1.029			1.024		

Note: Unstandardized WLSMV (probit) estimates for paths, ^†^*p* ≤ 0.10; *p ≤ 0.05; **p ≤ 0.01; ***p ≤ 0.001

## Results

Only 11.9% of the women in the present sample had a secondary/university education, while the corresponding number among men was 26.9% (*p* < .001). Further, a higher percentage of women (32.3 vs. 20.0%*, p* < .001) reported that they had suffered from depression previously in life and/or had experienced chronic loneliness (13.7 vs. 6.8%, *p* < .001). A similar sex difference was observed for ‘period(s) of longstanding stress’, although this association did not reach statistical significance. Women’s ratings of their own self-esteem were generally lower than men’s (∆ = 0.6, *p* < .001), but they were somewhat more satisfied with their social situation (∆ = 0.2, *p* < .05). Finally, during the five time periods specified, a larger proportion of women (18.5%) than men (9.1%) developed dementia (*p* < .001) (Table 1).

### Confirmatory factor analyses

As noted above, five manifest indicators were assumed to reflect the latent variable *General psychological distress:* previous depression, stress, self-esteem, chronic loneliness, and satisfaction with social situation. When the hypothesized measurement model was fitted to the data, all indicators were shown to be significantly related to the latent construct. Factor loadings ranged between 0.491 (stress) and 0.669 (satisfaction with social situation). However, the fit indices for this model indicated poor fit (Table 2). In order to identify localized areas of misfit, we ran the model without the imputed data. Regarding factor loading size and significance, this analysis demonstrated similar results (not shown here, but can be requested from the first author). The modification indices obtained from this analysis suggested that two additional paths (both representing residual covariances) should be added to the model: (1) depression with stress and (2) satisfaction with social situation and self-esteem (for a more detailed discussion, see below). This significantly ameliorated model fit, as indicated by the following indices: χ^2^/df = 2.3, TLI (0.981), RMSEA (0.034) and WRMR (0.388). The final measurement model, with one latent construct comprising the covariance between these factor indicators as well as two residual covariances, was thus considered to demonstrate a good fit to the data. As anticipated, and indicated by the factor loadings, positive relationships were observed for distress and self-esteem (λ = 0.428, 95% CI [0.313–0.543]), as well as for distress and satisfaction with social situation (λ = 0.540, 95% CI [0.420–0.660]) (both variables were coded from 1 ‘good’ to 7 ‘bad’). Likewise, positive relationships were observed for depression (λ = 0.601, 95% CI [0.475–0.728]), stress (λ = 0.412, 95% CI [0.268–0.555]) and chronic loneliness (λ = 0.696, 95% CI [0.557–0.835]).

### Full structural models

**Fig. 1:**
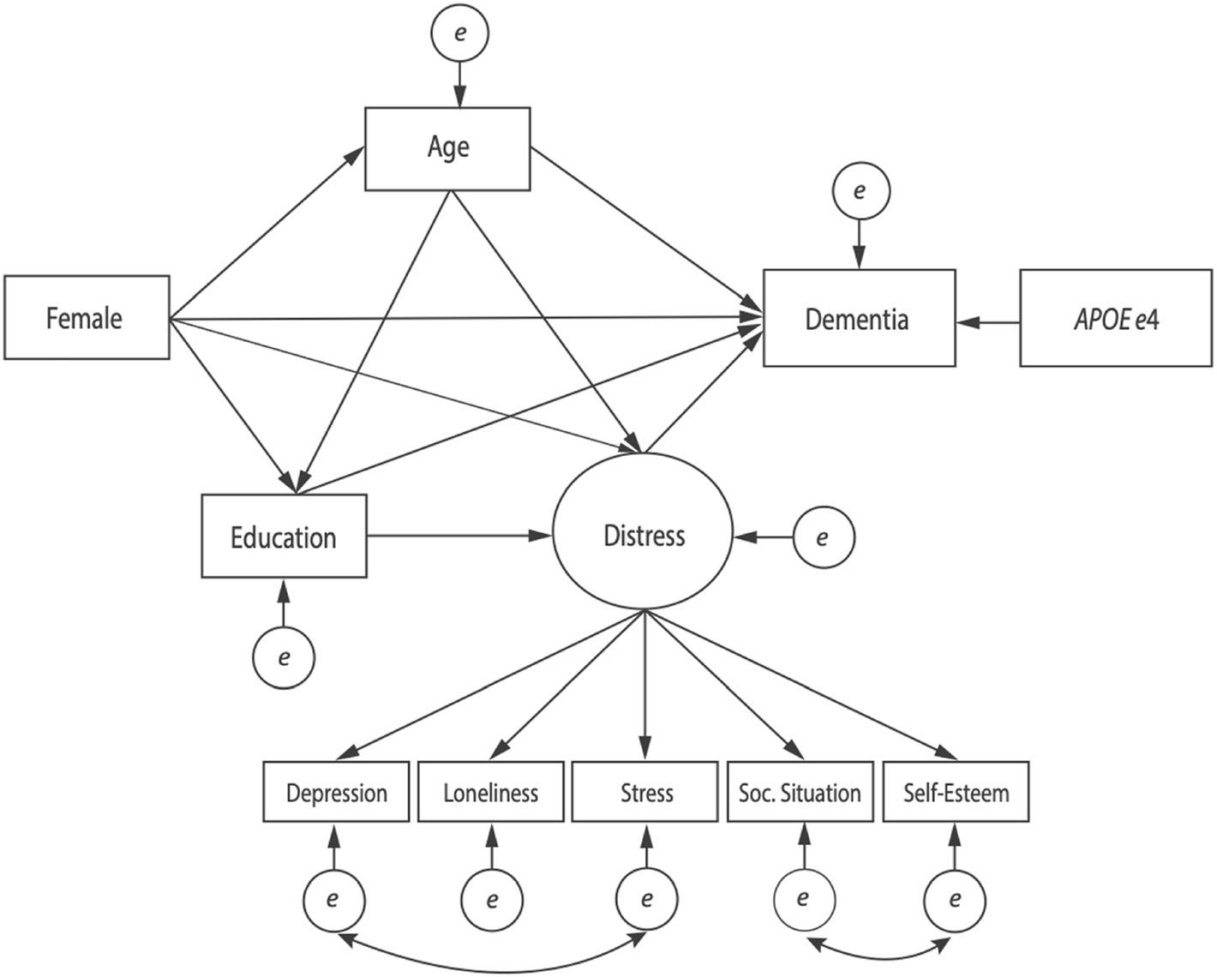
Conceptual model. Testing whether the effect of female sex on dementia is mediated by differences in 1) educational attainment and 2) general psychological distress

The conceptual SEM model (Fig. 1) illustrates the hypothesized relationships between education, sex, general psychological distress and dementia and thus represents the first model that was tested (Table 3, M1). As shown in the figure, female sex was assumed to have both indirect effects, via distress and education, and a direct effect on dementia. Education was also expected to have a direct effect on dementia. Additionally, indirect paths from female sex to dementia via age (measured at baseline) were specified, as the women in our sample were, on average, older than the men (all men born in 1930, women born in 1908, 1914, 1918, 1922 or 1930). Age was also hypothesized to have a direct effect on dementia as well as indirect effects via education and distress. *APOE* ɛ4 allelic status was assumed to only have a direct effect on the outcome variable. Finally, the residual covariances identified through the confirmatory factor analysis were both included.

**Fig. 2:**
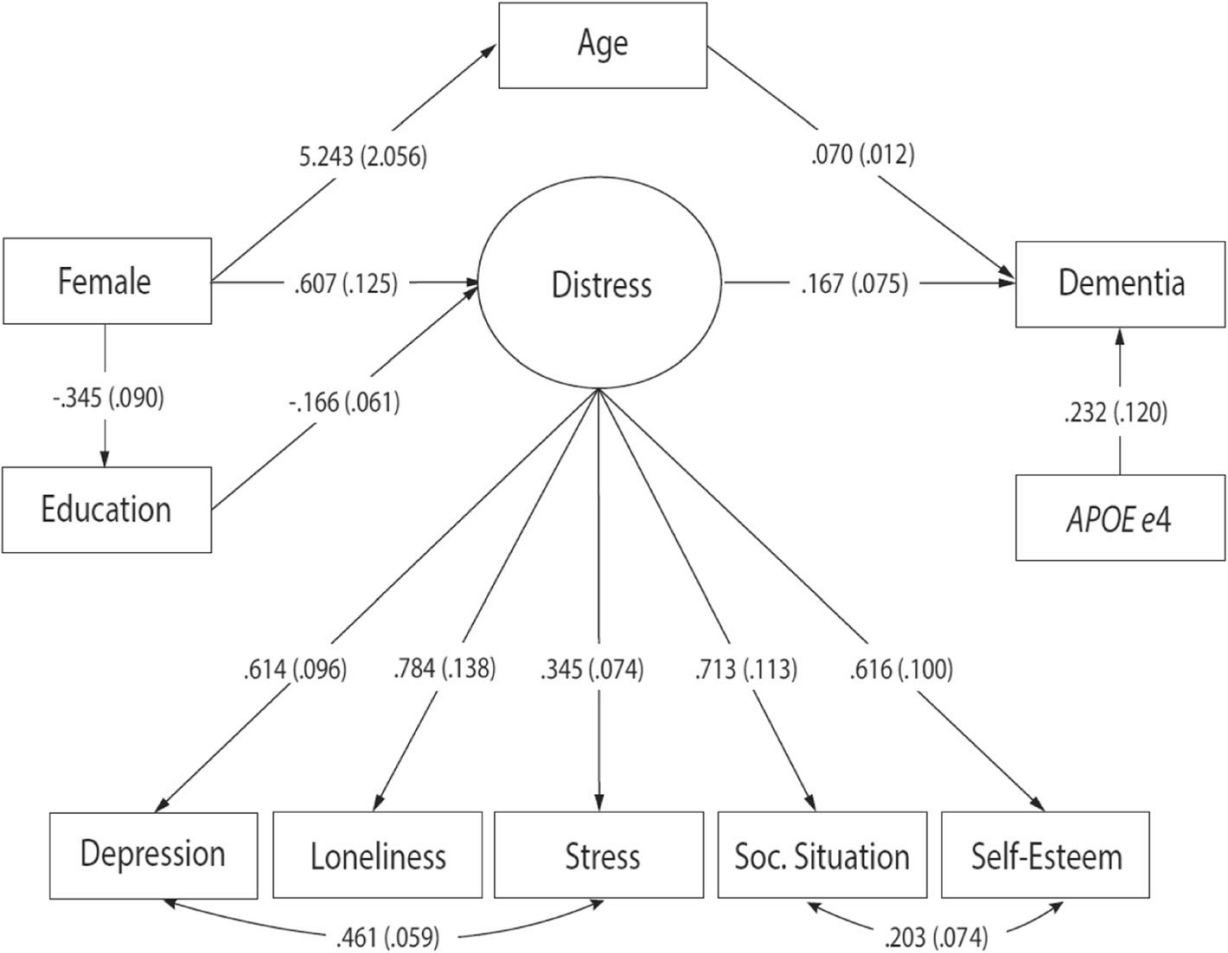
Final structural model. Unstandardized WLSMV (probit) estimates. Standard errors in parentheses

This model was tested on a sample of 861 individuals and all observed dementia cases occurred during or after 2002 (*n* = 144). All paths, except those representing the direct effects of education and female sex on age and dementia as well as the effects of age on education and distress, were, very close to, or statistically significant (Distress → Dementia: β = 0.150, 95% CI [− 0.003–0.302]; *APOE* ε4 → Dementia: β = 0.231, 95%CI [− 0.003–0.465]) (Table 3, M1). The total effect of female sex on dementia was estimated at 0.476 (95% CI [0.197–0.754]) (Table 4). Overall, the fit indices (Table 3, M1) were considered indicative of acceptable fit. Because ‘final models in SEM should represent the best fitting, albeit most parsimonious model’ (i.e., all parameters should be statistically significant), non-significant paths were removed one by one, starting with the path with the highest *p*-value [79]. After these respecifications, the following fit indices were obtained (Table 3, M2): χ^2^/df = 2.4, TLI = 0.921, RMSEA = 0.041 and WRMR = 1.029. The χ^2^/df ratio was below the recommended value of 3 [87] and TLI was above .90 [92]. RMSEA was below the suggested cut-off of .05 [88]. WRMR was not below 1, but because it is known to be sensitive to non-normality and larger sample sizes [90], this was judged to be of minor importance. Taken together, this model was considered to demonstrate a good fit to the data and no further respecifications were made. The final model is illustrated in Fig. 2.

**Table 4:** Direct, indirect, and total effects of female sex on dementia

	M1 (conceptual, t_1_)			M2 (final, t_1_)		
	β	95% CI	*p*	β	95% CI	*p*
Total	0.476	0.197–0.754	***	0.477	0.199–0.756	***
Direct	0.008	− 1.310 – 1.011	ns.	–	–	–
Indirect	0.468	− 0.845 – 1.466	ns.	0.477	0.199–0.756	***
Specific indirect						
Via age	0.361	− 0.631 – 1.353	ns.	0.366	0.076–0.657	**
Via distress	0.086	− 0.011 – 0.183	†	0.101	0.003–0.200	*
Via education	0.007	− 0.036 – 0.050	ns.	–	–	–
Via education → distress	0.008	− 0.004 – 0.019	ns.	0.004	− 0.003 – 0.022	ns.
Via age → education	0.001	− 0.005 – 0.007	ns.	–	–	–
Via age → distress	0.005	− 0.015 – 0.024	ns.	–	–	–
Via age → education → distress	0.001	− 0.003 – 0.024	ns.	–	–	–

Note: Unstandardized WLSMV (probit) estimates, ^†^*p* ≤ 0.10; *p ≤ 0.05; **p ≤ 0.01; ***p ≤ 0.001

An inverse relationship was observed between female sex and education (β = − 0.345, 95% CI [− 0.521- -0.170), and a positive association was detected between sex and age (β = 5.243, 95% CI [1.213–9.273]). The effects of *APOE* ɛ4 (β = 0.232, 95% CI [− 0.002–0.466]) and age (β = 0.070, 95% CI [0.047–0.093]) on dementia were also positive, indicating risk increase. Level of distress was shown to be predicted by both female sex (β = 0.607, 95% CI [0.361–0.852) and education (β = − 0.166, 95% CI [− 0.286- -0.047]) and, in turn, observed to be positively and significantly associated with dementia (β = 0.167, 95% CI [0.020–0.314]). Finally, the total effect of female sex on dementia was estimated at 0.477 (95% CI [0.199–0.756]), and the indirect effects via age (β = 0.366, 95% CI [0.076–0.657) and distress (β = 0.101, 95% CI [0.003–0.200]) were significant, whereas the indirect effect via education and distress (β = 0.004, 95% CI [− 0.003–0.022]) was not (Table 4).

Lastly, we tested whether this model would hold also when excluding dementia cases, starting with those that occurred in closest proximity to baseline, i.e., with individuals who were diagnosed before 2003 (t_2_). While the effects of sex and education on distress remained similar in size and significance, the effect of distress on dementia did not (β = 0.090, 95% CI [− 0.057–0.238]). The fit indices for this model were practically identical to those obtained for M2 (Table 3, M3). To investigate whether the effect size would decrease even more when further extending time from baseline to diagnosis, we also tested the model at t_3_ (excluding dementia cases that occurred before 2004), t_4_ (excluding dementia cases that occurred before 2005), and t_5_ (excluding dementia cases that occurred before 2006). The overall results (not shown here, but can be requested from the first author) remained virtually unchanged compared to Model 1 and 2. The following effects of distress on dementia were estimated at t_3_: β = 0.079, 95% CI [− 0.073–0.232], t_4_: β = 0.046, 95% CI [− 0.117–0.209], and t_5_: β = 0.085, 95% CI [− 0.079–0.248].

## Discussion

The present study sought to further explore the multifaceted relationship between dementia and social inequity by using structural equation modelling to test whether general psychological distress and educational attainment mediate the association of sex with disease risk. We also conducted a sensitivity analysis to investigate the possibility of reverse causality. Overall, the results suggest that female sex predicts distress, both directly and indirectly via educational attainment, and that distress could increase the risk of dementia. The latter association did not, however, remain significant when time from baseline to diagnosis was increased, implying that reverse causality bias cannot be definitely ruled out at this stage.

Many influential dementia risk factors, e.g., stress, depression and social isolation, are known to be interrelated and to follow social gradients – in favour of men and individuals with higher SES [41–47]. Thus, confirmatory factor analysis was used initially to corroborate the hypothesized relationships between five manifest indicators (previous depression, stress, chronic loneliness, low self-esteem and satisfaction with social situation) of *General psychological distress* (Table 2). As expected, this analysis confirmed that distress has an adverse impact on individuals’ mental health and self-esteem as well as on their general sense of belonging. As stated above, two residual covariances were added at this stage of the modelling procedure – 1) depression with stress and (2) satisfaction with social situation and self-esteem – demonstrating that these indicators have something in common that goes beyond their association with general distress.

In a second step, a full structural model was fitted to the data to test the main hypotheses, i.e., that 1) the effect of female sex on dementia is mediated directly by differences in level of educational attainment, and 2) that the effect of female sex on dementia is mediated directly, as well as indirectly via education, by level of general psychological distress, also after controlling for age and *APOE* ε4 allelic status. Results from the conceptual model indicated that, contrary to our expectations, no direct effects of female sex and education on dementia could be observed [cf. 16, 18, 19, 22]. Thus, our findings did not provide support for the first hypothesis (H_1_), even though low educational attainment was, as expected, predicted by female sex (Table 3, M1). While the empirical evidence provided here is not sufficient to draw that conclusion, we suspect that the lack of significant direct effects could be the result of unmeasured interaction effects, rather than an expression of full mediation. Particularly since *APOE* ε4 has previously been suggested to moderate the effect of education [78, 94] and that its effect might, in turn, be moderated by sex [95]. Still, in more general terms, the results underline the importance of attending to education as a ‘gendered’ dementia risk factor [16, 20, 66]. Results from the final model provided partial supported for the second hypothesis (H_2_) (Table 3, M2 and Fig. 2). First, significant paths were observed between sex and level of distress as well as between education and distress. In line with a range of previous studies, this suggests that women, and individuals with lower education, are more likely to suffer from poor mental health [41–44, 53]. Second, a significant path was observed between distress and dementia. The succeeding computation of total and indirect effects indicated that while the specific indirect effect of female sex on dementia via level of general psychological distress was significant (though relatively small in size), the indirect effect represented by the paths female sex → education → distress → dementia, was not (Table 4). Considering that social gradients exist in relation to many of the manifest indicators included in the latent construct, e.g., depression, stress and social isolation, and that these factors have previously been suggested to increase the risk of dementia [36, 37, 50, 55, 57, 59], these results were in line with our overall expectations.

As stated above, the present study addresses the issue of reverse causality and a range of measures were taken to limit the possibility of such bias. Nevertheless, when we restricted the sample to include only individuals who were diagnosed during or after 2003, the effects of sex and education on distress remained unaffected, while the effect of distress on dementia decreased and was no longer significant. Despite this, we subsequently increased time from baseline to diagnosis even further to investigate whether the effect size would continue to decrease over time. This was done by sequentially excluding dementia cases that occurred before 2004 (t_3_), 2005 (t_4_) and 2006 (t_5_). Interestingly, the estimated effects of distress on dementia decreased marginally at the first two time points, but increased slightly at the third (compared to that estimated at t_2_). Taken together, and while the overall decline in effect size would suggest a case for reverse causality, we argue that at least four methodological aspects must be taken into consideration before definitely drawing that conclusion. First, the loss of significance could, at least in part, be the result of the reduction in dementia cases that occurred when time to diagnosis was increased. Second, it is possible that the effect diminished because of selection, i.e., that the longer an individual survives without developing dementia, the healthier s/he is, also in terms of mental health, meaning that strength of previously observed associations are weakened as the cohort ages [29, 96]. It is also worth noting that the effect never diminished altogether and was of similar magnitude at t_2_ (0.090) and t_5_ (0.085), despite the substantial reduction in dementia cases (see Table 1). The third aspect is related to one of the primary virtues of structural equation modelling, namely that the latent construct comprises *only* the covariance between the indicators (as opposed to an additive index). In practice, this implies that because some of the factor indicators refer to conditions earlier in life, having experienced symptoms of distress only at baseline, i.e., in close proximity to diagnosis, is not enough for an individual to score high on the latent factor *‘General psychological distress’*.

### Strengths and limitations

The principal strengths of the present study are its methodological approach, which enables the investigation of hypothesized ‘causal structures’ [79], and its possible contribution to the discussion of potential mechanisms linking gender inequity to dementia risk. Additional strengths are the prospective population design, the extensive neuropsychiatric examinations through which dementia was diagnosed, and the attempt to explicitly address reverse causality bias by stratifying the time from baseline to diagnosis. The main limitations are the fairly short follow-up time and the relatively low number of dementia cases. Because the majority of individuals in our sample were born in 1930, the sample only includes a small proportion of information from individuals above age 82, which partly explains why the number of dementia cases is still limited. Further, due to the low number of dementia cases, we were not able to distinguish between dementia subtypes in the present study. Although most people with dementia have brain abnormalities that can be attributed to more than one cause [1, 3–7], this is a limitation in the sense that the mediating risk factors linking, e.g., sex to disease risk may vary by subtype [1, 13–17]. Finally, the quality of the factor indicators must be acknowledged. First, they are all self-reported, which means that the information on, e.g., previous depression is not based on clinical data. This is a possible limitation, inter alia, in the sense than women might be more prone to reporting mental ill health or symptoms thereof.

## Conclusions

The present study found that social (dis) advantage, here indicated by female sex and low educational attainment, predicts general psychological distress in older individuals without dementia, regardless of whether it should be regarded as a risk factor or pre-diagnostic sign. Nevertheless, it cannot be concluded with certainty that distress mediates the effects of female sex on dementia, although this hypothesis was partly supported by the data. Therefore, the principal implication of these findings is that they underscore the importance of attending to both education and distress as ‘gendered’ phenomena when considering the nature of their associations with dementia - in future studies as well as in clinical practice. Also, given the well-established sex and socio-economic (including educational) differences in psychological well-being, and its potential influence on dementia risk, these results highlight the urgent need for longitudinal studies with long (er) follow-ups. This is crucial if we are to fully account for reverse causality bias and thereby enable a more nuanced understanding of how and why social inequity influences health, specifically dementia risk, late in life. Further investigation of the hypotheses tested is needed, and future studies of sex differences in dementia should also strive to account for the possibility of interaction effects – between structural disease determinants as well as between genetic risk factors and potentially mediating factors. Finally, we encourage forthcoming studies to also explore the significance of differences between men and women in the *timing* of risk factors (e.g., cardiometabolic diseases, which men usually develop earlier than women) since they could give rise to sex-specific selection effects [16].

## Data Availability

The data that support the findings of this study are available from the Sahlgrenska Academy, Institute of Neuroscience and Physiology (Neuropsychiatric Epidemiology Unit), but restrictions apply to the availability of these data, which were used under license for the current study, and so are not publicly available. Data are however available from the authors upon reasonable request and with permission of PI, Ingmar Skoog.

## Abbreviations

AD: Alzheimer’s disease
*APOE*: Apolipoprotein E
CFA: Confirmatory Factor Analysis
DSM-III-R: Diagnostic and Statistical Manual of Mental Disorders, 3rd Edition Revised
MMSE: Mini Mental State Examination
RMSEA: Root Mean Square Error of Approximation
SEM: Structural Equation Modelling
SES: Socio-Economic Status
TLI: Tucker Lewis Index
VaD: Vascular Dementia
WLSMV: Weighted Least Squares Means and Variance Adjusted
WRMR: Weighted Root Mean Square Residual
